# Supplementary material for: Recurrent patterns of DNA copy number alterations in tumors reflect metabolic selection pressures
Source: Mol Syst Biol. 2017 Feb 15;13(2):914. doi: 10.15252/msb.20167159 (PMC5327725; doi:10.15252/msb.20167159)
Supplement: Supplementary file 1 — Appendix [file MSB-13-914-s001.pdf]

Appendix

Recurrent patterns of DNA copy number alterations in tumors reflect metabolic selection pressures

Nicholas A Graham†, Aspram Minasyan†, Anastasia Lomova, Ashley Cass, Nikolas G Balanis, Michael Friedman, Shawna Chan, Sophie Zhao, Adrian Delgado, James Go, Lillie Beck, Christian Hurtz, Carina Ng, Rong Qiao, Johanna ten Hoeve, Nicolaos Palaskas, Hong Wu, Markus Müschen, Asha S Multani, Elisa Port, Steven M Larson, Nikolaus Schultz, Daniel Braas, Heather R Christofk‡, Ingo K Mellinghoff‡, and Thomas G Graeber*

† equal contribution, ‡ equal contribution, * corresponding author, correspondence to: tgraeber@mednet.ucla.edu

Running title: Tumor CNA reflect metabolic selection

Table of Contents:

Appendix Figures S1-11 ..... pp. 2-21
Appendix Table S1 ..... p. 22
Appendix References ..... p. 23

Copy number profiling data. Wild-type and genetically modified MEF samples and FDG-PET-imaged human breast tumors (GEO accession GSE63306).

Interactive website for user-defined cross-tumor type and cross-species CNA conservation analysis: to perform analysis analogous to that in Figures 1C, 2D, and 4E using any combination of tens of available CNA signatures from human tumors and mouse models (and additional signatures as they become available) and/or the inclusion of uploaded CNA signatures. http://systems.crump.ucla.edu/cna_conservation/.

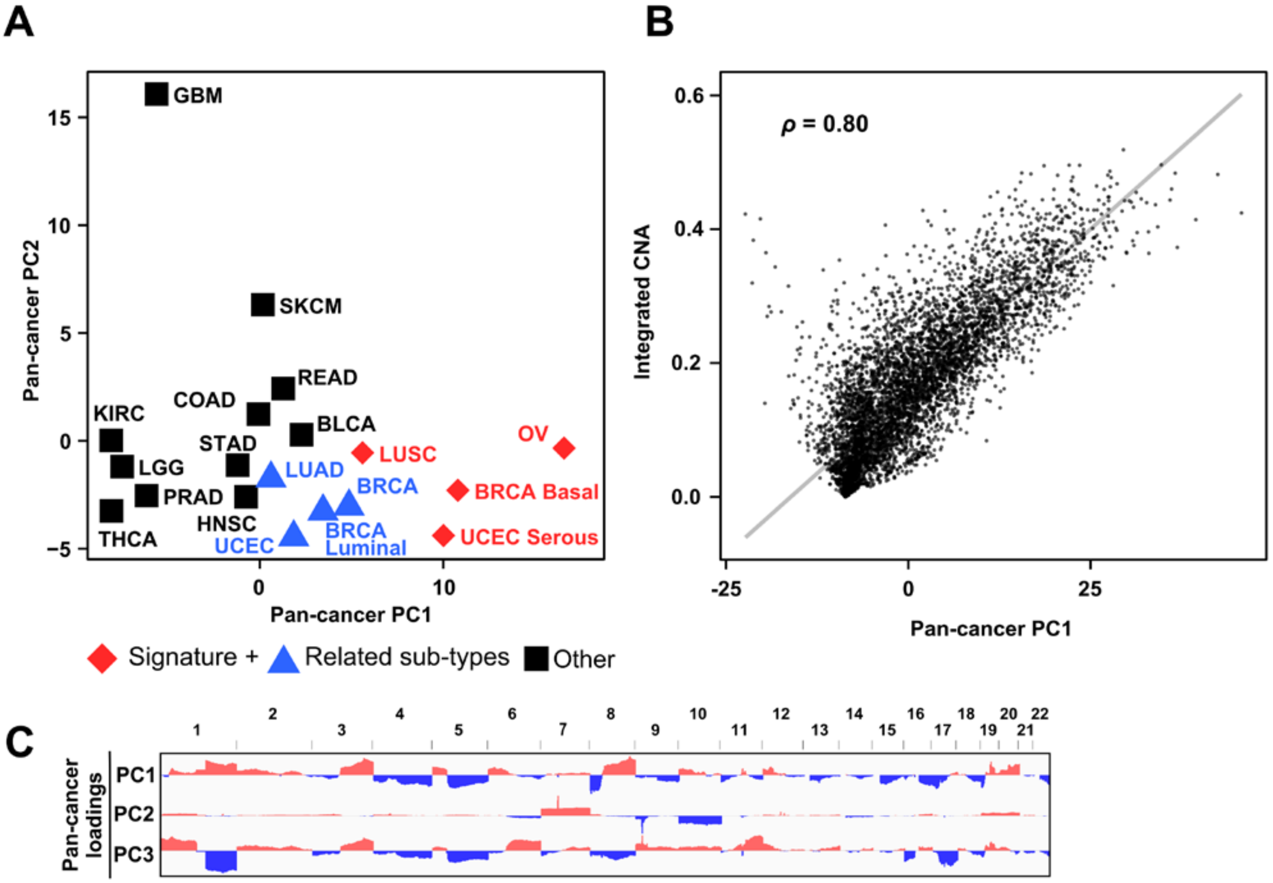

D

Tumor type	Pan-cancer PC1 (Sig. A strength)	Pan-cancer PC2	Tumor-type specific PC1 extremes	Tumor-type specific PC2 extremes
OV	16.6		Sig A vs. Sig B' (both Sig A like)	aneuploid vs. diploid
LU	5.3		Sig A vs. Sig B''	aneuploid vs. diploid
BRCA	4.9		Sig A vs. Sig B'''	aneuploid vs. diploid
BLCA	2.3		sub-signatures	aneuploid vs. diploid
UCEC	1.8		Sig A vs. diploid	-
READ	1.3		aneuploid vs. diploid	sub-signatures
SKCM	0.2	6.3	aneuploid vs. diploid	sub-signatures
COAD	-0.1		aneuploid vs. diploid	sub-signatures
HNSC	-0.7		aneuploid vs. diploid	sub-signatures
STAD	-1.2		aneuploid vs. diploid	sub-signatures
GBM	-5.6	16.1	'Chr 7 amp/Chr 10 del' vs. diploid	-
PRAD	-6.2		aneuploid vs. diploid	sub-signatures
LGG	-7.5		sub-signatures	-
THCA	-8.1		mild aneuploid vs. diploid	-
KIRC	-8.1		-	-

Tumor type	Subtype	Signature type	Pan-cancer PC1		Pan-cancer PC3
			All	Sig. A / Sig. B	
OV	proliferative	Sig A	16.6	20.6	
	mesenchymal	Sig B' (Sig A like)		12.6	
LU	LUSC	Sig A	5.3	5.6	↑ pan PC3
	LUAD	Sig B''		0.6	↓ pan PC3
BRCA	Basal	Sig A	4.9	10.8	↑ pan PC3
	Luminal	Sig B'''		3.4	↓ pan PC3
UCEC	Serous	Sig A	1.8	10.0	
	Non-serous	diploid		-1.0	

Appendix Figure S1. *Summary of pan-cancer PCA trends and tissue-specific PCA trends.* Related to Fig. 1.

Pan-cancer PCA summary: Pan-cancer PC1 scores primarily separate diploid from highly aneuploid tumors (panel B), while pan-PC2 scores primarily separate glioblastoma (GBM) from the other tumor types (panel A). Pan-PC3 tends to separate Sig A BRCA and LU tumors from Sig B BRCA and LU tumors (panel C and Fig. 1A).

(A) PC1 versus PC2 for pan-cancer PCA of copy number data from a balanced, random sampling of tumors of 15 tumor types from The Cancer Genome Atlas (TCGA). The average tumor PC scores for each tumor subtype are shown. PC2 distinguished GBM from the other tumor types. The GBM-associated PC2 loadings are chromosome 7 high and focally high for the chr. 7 *EGFR* locus. Additionally, pan-cancer PC2 loadings are low for chr. 10 including the *PTEN* locus, and focally low near the chr. 9 *CDKN2A* locus. These events reflect the major CNA events of GBM (Ohgaki & Kleihues, 2007). PC1 versus PC3 is shown in Fig. 1A.

(B) Pan-cancer PC1 scores refect the degree of aneuploidy as measured by the Integrated CNA score. Spearman rank correlation indicated.

(C) The pan-cancer PCA loadings for PC1-3 are shown with respect to their genomic position for a graphical representation of the main trends found by PCA analysis. The pan-cancer PCA signatures (gene loadings) and tissue-specific PCA signatures are available at the interactive web-interface resource (http://systems.crump.ucla.edu/cna_conservation/).

(D) Tissue-specific PCA analysis tended to either separate diploid from aneuploid tumors, or define sub-signatures at the extreme ends of the PC scores – as exemplified by the BRCA case of defining signature A and signature B tumors shown graphically in Fig. 1B. Most tumor types had both a diploid to aneuploid principal component and a sub-signatures principal component, as either PC1 or PC2. Additional information of how different tumor types are related to the signature A and B sub-types is summarized in the hierarchical cluster results (Fig. EV3 and Appendix Fig. S3).

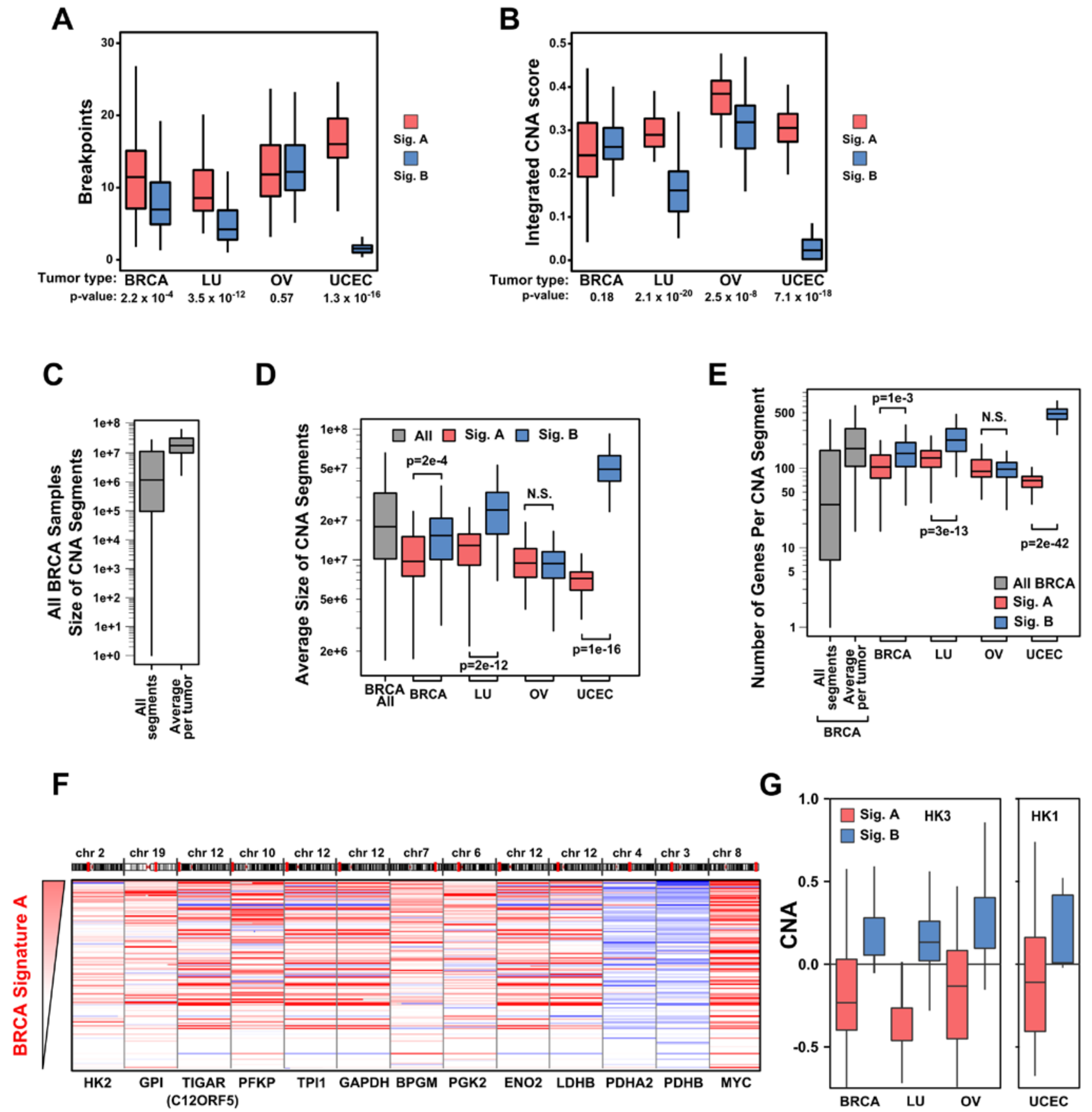

Appendix Figure S2. *Characterization of the principal component analysis-defined human tumor CNA signatures.* Related to Fig. 1.

(A-B) PCA-defined signature A tumors exhibited more genomic breakpoints (A) and a higher degree of copy number alterations (integrated CNA score) (B) than signature B tumors. The exceptions are ovarian (OV), which exhibited similar numbers of genomic breakpoints in signature A and B tumors, and breast (BRCA), which exhibited similar levels

of copy number alterations in signature A and B tumors. Mann-Whitney U-test p-values are shown for each signature A versus signature B comparison. Data is presented in box (median, first and third quartiles) and whisker (extreme value) plots.

(C) The distribution of sizes in base pairs of CNA segments for BRCA as determined using two different methods. 'All segments' represents all individual segments from all BRCA tumors. 'Average per tumor' represents the distribution of per tumor average segment sizes.

(D-E) Signature A tumors exhibited smaller CNA segments (D) and fewer genes per CNA segment (E) than signature B tumors. The exception is OV, which displayed similar segment size and gene number per segment in both signature A and B. For comparison, the distribution of segment sizes across all BRCA tumors is shown in panel D (BRCA All). Likewise shown in panel E are the distribution of number of genes per CNA segment using all individual segments across all BRCA tumors, and using the per tumor average. Mann-Whitney U-test p-values are shown for each Sig. A versus Sig. B comparison. The conversion factor between segment size and number of genes is approximately 1 gene per 100,000 base pairs.

(F) The PCA-identified signatures were defined by genome-wide patterns, as single gene loci were not consistently altered in all tumor cases with a strong signature score, here shown for glycolysis-related genes in the PC1-sorted signature A breast tumors from Figure 1B.

(G) Signature B tumors amplify HK3 (BRCA, LU, OV) and HK1 (UCEC) showing alternative HK amplification as compared to signature A tumors (which amplify HK2).

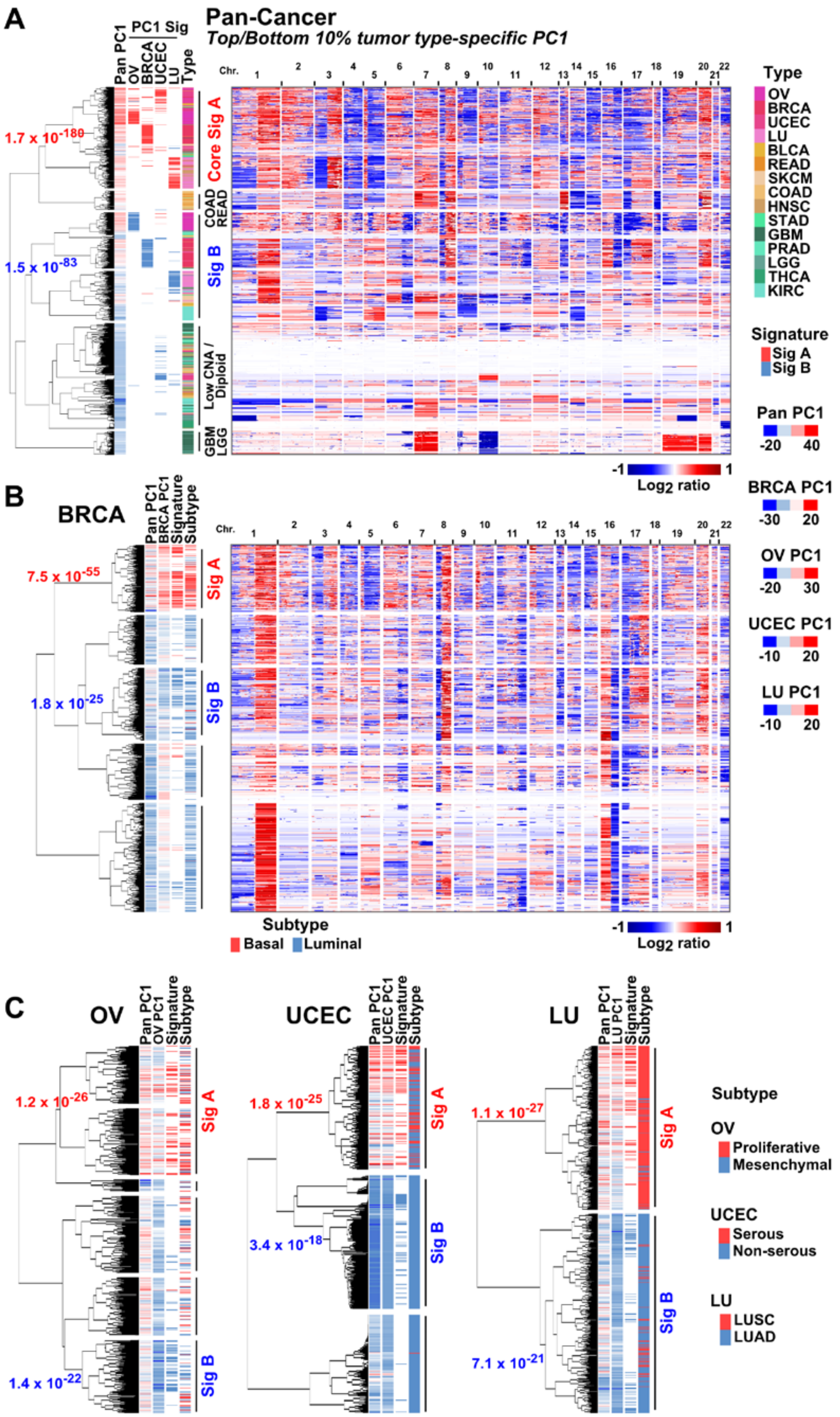
A
Pan-Cancer
Top/Bottom 10% tumor type-specific PC1
Pan PC1
PC1 Sig
OV
BRCA
UCEC
LU
Type
Chr.
1.7 x 10-180
1.5 x 10-83
Core Sig A
COAD READ
Sig B
Low CNA / Diploid
GBM LGG
Type
OV
BRCA
UCEC
LU
BLCA
READ
SKCM
COAD
HNSC
STAD
GBM
PRAD
LGG
THCA
KIRC
Signature
Sig A
Sig B
Pan PC1
-20
40
-1
1
Log2 ratio
B
BRCA
Pan PC1
BRCA PC1
Signature
Subtype
7.5 x 10-55
1.8 x 10-25
Sig A
Sig B
BRCA PC1
-30
20
OV PC1
-20
30
UCEC PC1
-10
20
LU PC1
-10
20
Subtype
Basal
Luminal
Log2 ratio
C
OV
UCEC
LU
OV PC1
UCEC PC1
LU PC1
1.2 x 10-26
1.4 x 10-22
1.8 x 10-25
3.4 x 10-18
1.1 x 10-27
7.1 x 10-21
Subtype
OV
Proliferative
Mesenchymal
UCEC
Serous
Non-serous
LU
LUSC
LUAD

Appendix Figure S3. *Hierarchical clustering confirms the existence of the shared pan-cancer CNA signatures across multiple tumor types, and distinct signature subtypes within tumor types.* Related to Figs. 1 and EV3.

(A) Pan-cancer clustering of 1,171 copy-number profiles using tumor type-specific PCA-based signatures from 15 tumor types. PCA analysis was performed on each tumor type individually (as in Fig. 1B), and the top 10% and bottom 10% PC1 score-based tumors were used in the clustering step. The dendrogram reveals a multi-tumor cluster highly enriched in core signature A tumors (hypergeometric p-value=$1.7x10^{-180}$) and a cluster enriched in signature B tumors (hypergeometric p-value=$1.5x10^{-83}$). This result demonstrates how tumor type-specific PCA analysis applied individually to BRCA, OV, UCEC, LU and other tumors identified pan-cancer tumors (those with high tumor-type specific PC1 scores) that share a highly related signature (Signature A). In the key, the individual tumor types and pan-cancer PC1 scores are indicated. Additionally, for the core tumor types (BRCA, OV, UCEC, LU), tumor type-specific PC1-based signatures (Sig. A and Sig. B, top and bottom 10% based on PC1 scores) are indicated. The gene loci CNA levels in the heatmap are ordered based on their chromosomal locations (columns).

(B) Clustering of 873 BRCA copy-number profiles. The dendrogram reveals a distinct cluster enriched in BRCA signature A tumors (hypergeometric p-value=$7.5x10^{-55}$) as well as a cluster enriched in BRCA signature B tumors (hypergeometric p-value=$1.8x10^{-25}$). Similar to tumor type-specific PCA analysis (Fig. 1B), clustering differentiates between the basal and luminal BRCA subtypes.

(C) Clustering of 583 OV, 492 UCEC, and 727 LU copy-number profiles, respectively. The dendrograms reveal distinct clusters enriched in signature A tumors (hypergeometric p-values: OV=$1.2x10^{-26}$, UCEC=$1.8x10^{-25}$, LU=$1.1x10^{-27}$) as well as clusters enriched in signature B tumors (hypergeometric p-values: OV=$1.4x10^{-22}$, UCEC=$3.4x10^{-18}$, LU=$7.1x10^{-21}$). Similar to tumor type-specific PCA analysis (Fig. EV2A-C), clustering differentiates between the proliferative and mesenchymal subtypes in OV, serous and non-serous subtypes in UCEC, and LUSC and LUAD subtypes in LU.

(B-C) In the keys, tumor type-specific and pan-cancer PC1 scores are indicated. Additionally, tumor type-specific PC1-based signatures (Sig. A and Sig. B, top and bottom 10% based on PC1 scores) are indicated.

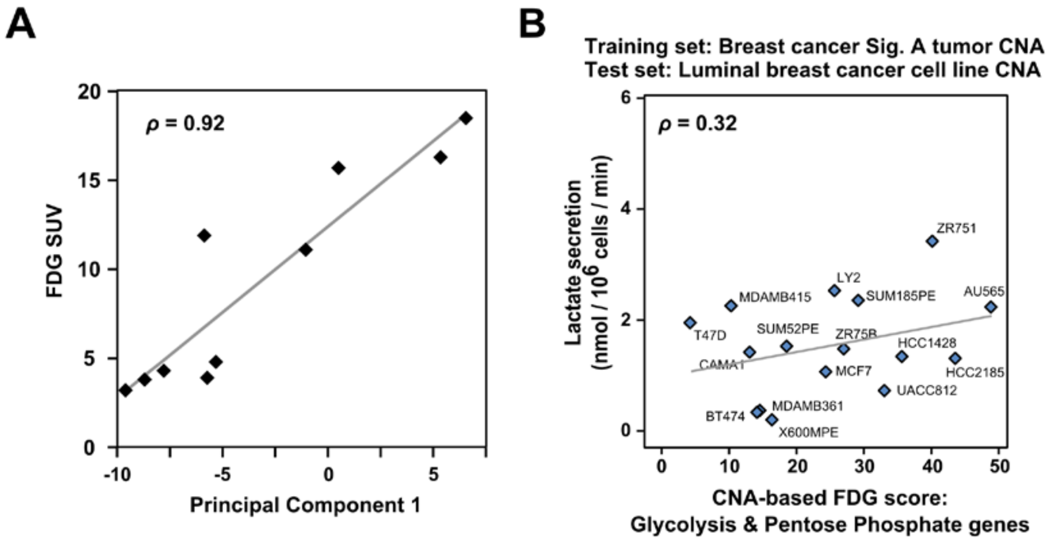

Appendix Figure S4. <u>*PCA-based CNA signatures are predictive of breast cancer glycolytic metabolism*</u> <u>in vivo</u> <u>*and breast cancer cell line metabolism*</u> <u>in vitro</u>. Related to Fig. 3.

(A) FDG uptake values in FDG-PET measured tumors are highly correlated with PC1 scores of a balanced, random sampling of tumors from the nine expanded signature A tumor types (defined in Fig. 2A) (ρ, Pearson rho correlation; p-value = 2 x 10^{-4}).

(B) CNA signature A-based WGV predictions of lactate secretion in luminal breast cancer cell lines. Luminal sub-type classifications are from published gene expression-based classification (Neve *et al*, 2006). The breast cancer-based glycolysis and pentose phosphate pathway (G & PP) WGV predictions are shown as a representative case. The resulting correlation (ρ = 0.32) is less than the case when CNA patterns are used to predict lactate secretion in basal breast cancer lines (ρ = 0. 71, Fig. 3E), further supporting a stronger relationship between signature A and the basal subtype of breast tumors.

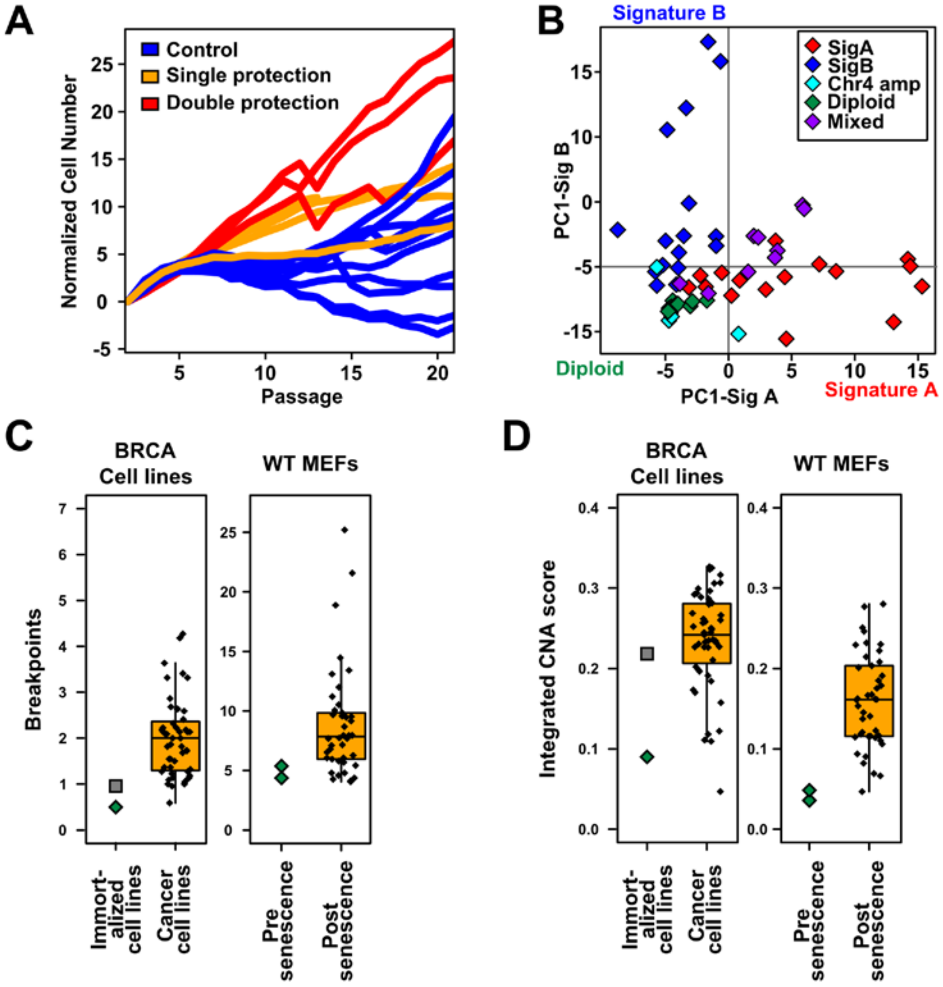

Appendix Figure S5. *Characterization of copy number alteration patterns from the mouse embryonic fibroblast (MEF) immortalization system.* Related to Fig. 4.

(A) Growth curves showing that protection of CD1 wild-type MEF lines from oxidative stress rescues cells from replicative senescence. Single protection: cells were cultured under physiological oxygen conditions (3% O_2) or by media supplementation with 250 U/ml of the ROS scavenging enzyme catalase. Double protection: cells were cultured at 3% O_2 and supplemented with catalase. Exogenous MYC expression also resulted in rescue from replicative senescence (not shown) and did not exclude evolution towards either signature A or B (Fig. 4A).

(B) In order to obtain pure signatures for A and B lines, we ran PCA individually on either signature A only or signature B only wild-type MEF lines (PC1-Sig. A and PC1-Sig. B, respectively). Samples not run in a particular analysis were projected onto the rotated PCA axes to allow for a full comparison. Plotting signature A against signature B scores revealed that CNA patterns of these two groups were generally orthogonal, with the exception of a few 'mixed' signature

samples that had CNA characteristics of both signature A and B. A less frequent CNA pattern involving chromosome 4 amplification was also observed.

(C-D) Breakpoints (C) and integrated CNA scores (D) of wild-type MEF pre-senescence versus post-senescence/immortalized cell lines, compared to human breast immortalized versus cancer cell lines as a reference. For the breast cell lines, the immortalized lines are MCF10A (green diamond) and MCF12A (gray square) (Neve *et al*, 2006). 'Pre-senescence' denotes early passage MEF cells (green diamonds). The number of breakpoints for the BRCA cell lines is lower than that of the MEF cell lines at least in part because the resolution of the OncoBAC array used to measure CNA in the breast cell lines (Neve *et al*, 2006) was lower than that of the aCGH microarray used to measure MEF CNAs.

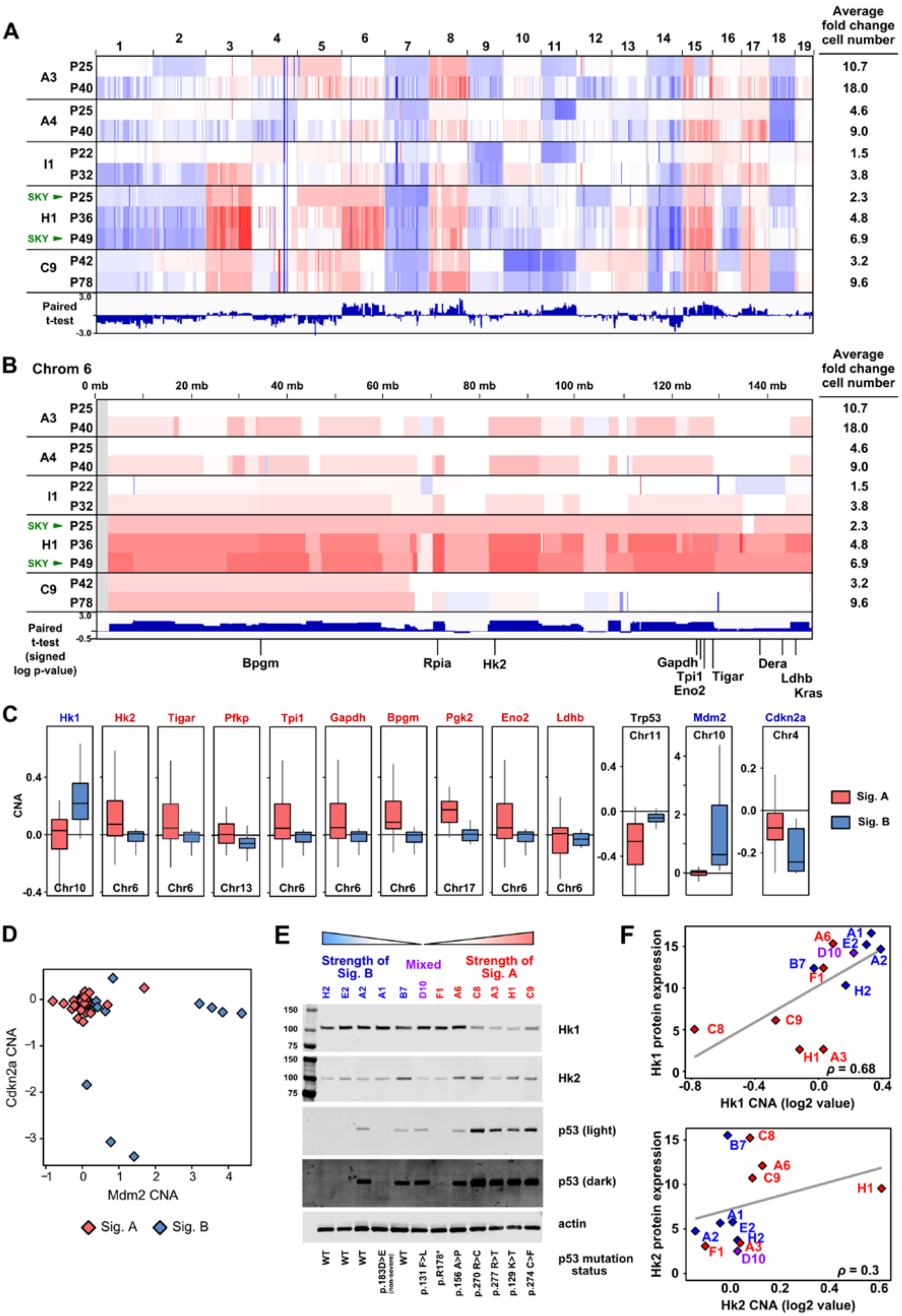
A
B
Chrom 6
C
D
E
F
Average fold change cell number
Paired t-test
Paired t-test (signed log p-value)
Bpgm
Rpia
Hk2
Gapdh
Tpi1
Eno2
Tigar
Dera
Ldhb
Kras
Sig. A
Sig. B
Cdkn2a CNA
Mdm2 CNA
Strength of Sig. B
Mixed
Strength of Sig. A
Hk1
Hk2
p53 (light)
p53 (dark)
actin
p53 mutation status
Hk1 protein expression
Hk1 CNA (log2 value)
Hk2 protein expression
Hk2 CNA (log2 value)
ρ = 0.68
ρ = 0.3

Appendix Figure S6. *A mouse embryonic fibroblast (MEF) immortalization system recapitulates the glycolysis gene-enriched CNA patterns observed in human tumors.* Related to Fig. 4.

(A-B) A paired t-test analysis of signature A MEF lines profiled at more than one passage number revealed genomic regions associated with mid- to late-passage CNA evolution ($\log_{10}$ t-test p-value signed positive for amplifications, negative for deletions; bottom row). The full genome (A) and chromosome 6 (B) are shown. As with the human tumor signatures A, this mid- to late-passage genomic signature was enriched for DNA amplifications of genes in the core glycolysis and glycolysis associated pathways and improved the enrichment of the Core Glycolysis pathway when added to the human and mouse models (Table EV2). We observed a general co-evolution of higher growth rates and increased CNA signature strength of MEF lines profiled at more than one passage number, as shown by the average fold change in cell number between 3T9 passages (see also Fig. 6D). The samples for which spectral karyotyping (SKY) was performed are indicated on the left by green arrows.

(C) Gene copy number alteration distributions of selected core glycolysis genes, *Tigar*, and of tumor suppressor p53 (*Trp53*) and the p53-associated cancer genes *Mdm2* and *Cdkn2a* in MEF lines.

(D) As in signature B BRCA human tumors (Fig. EV2E), amplification of *Mdm2* and deletion of *Cdkn2a* are mutually exclusive in signature B MEF cell lines.

(E) MEF lines representative of signature A and signature B (as defined by CNA profiles) were lysed and probed by immunoblotting for Hk1, Hk2 and p53. Signature A is associated with higher DNA copy number and higher protein expression of Hk2, whereas signature B is associated with higher CNA and protein levels of Hk1. Signature A cells, which are characterized by *p53* mutation (Appendix Table S1), show higher expression of p53 protein than signature B cells, which typically have wild-type p53. Mutations in p53 commonly lead to elevated protein levels via decreased degradation. Cell lines are arranged by their CNA-defined PCA signature strength. Actin was included as an equal loading control.

(F) Correlation between CNA values and relative intensity of protein expression for both Hk1 and Hk2. Western blots were quantified using ImageJ.

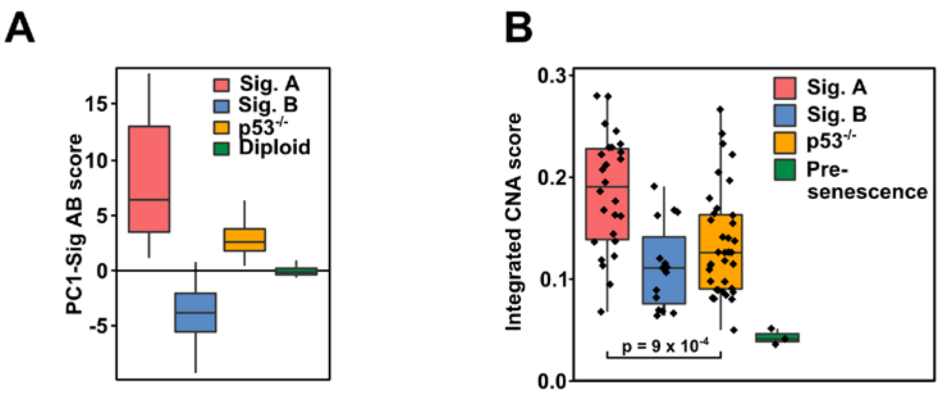

Appendix Figure S7. *Characterization of the p53-/- MEF CNA signatures.* Related to Fig, 4.

(A) CNA patterns of 37 p53-/- MEF samples from 29 independent sub-lines derived in standard 3T9 culture conditions resemble the wild-type MEF signature A CNA pattern, as demonstrated by positive PC1-Sig. AB scores when projected onto the wild-type MEF PC1 axis.

(B) p53-/- MEFs exhibit a lower degree of copy number alterations (integrated CNA score) than signature A MEFs. p53-/- cells do not undergo senescence and thus tend to have less strong copy number alterations.

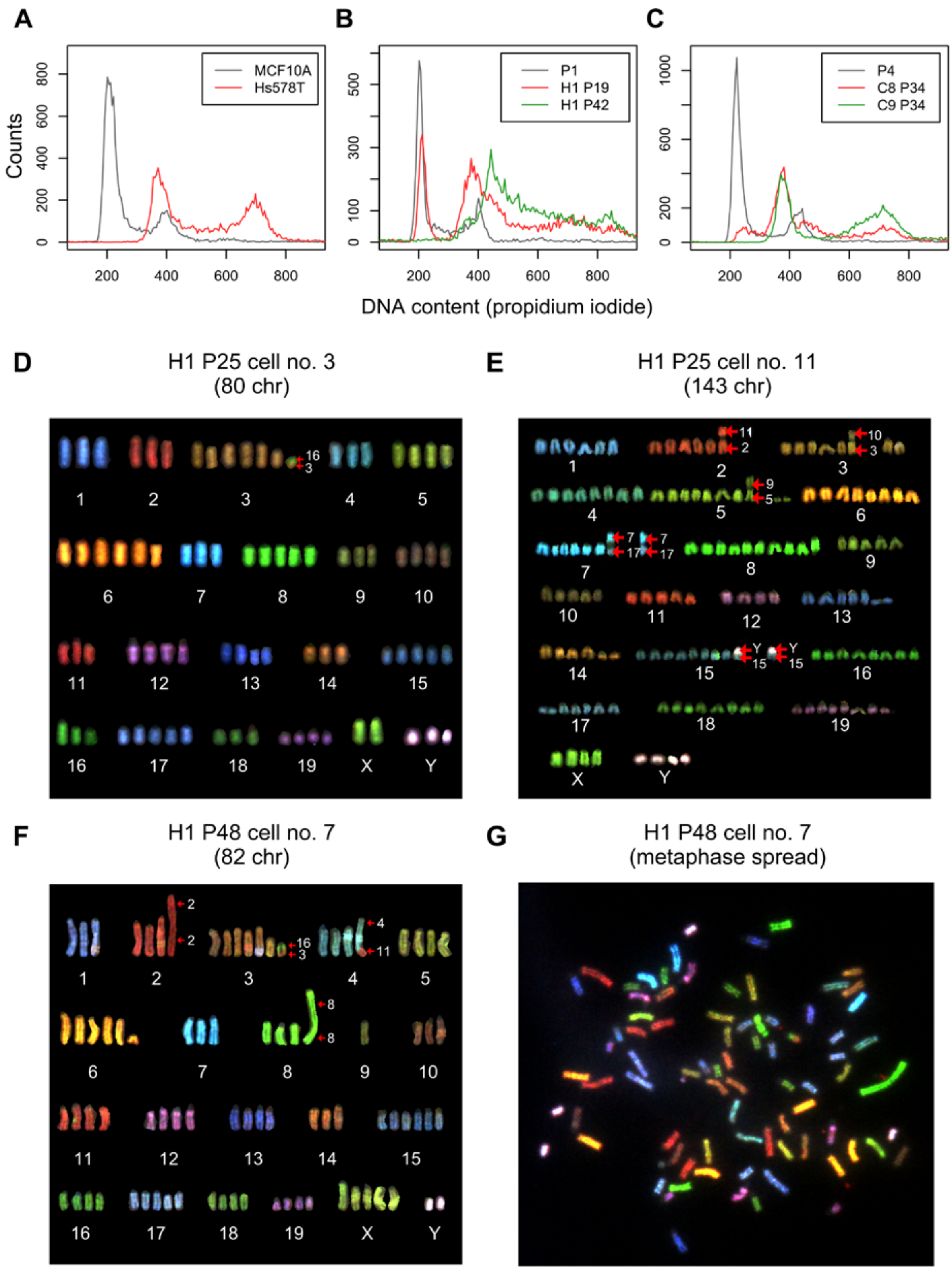
A
B
C
Counts
MCF10A
Hs578T
P1
H1 P19
H1 P42
P4
C8 P34
C9 P34
DNA content (propidium iodide)
D
H1 P25 cell no. 3
(80 chr)
E
H1 P25 cell no. 11
(143 chr)
F
H1 P48 cell no. 7
(82 chr)
G
H1 P48 cell no. 7
(metaphase spread)

H **H1 P25 (105 ± 36 chr)**

Cell no.	Chromosome count	Chromosomal abnormalities
1	147	del(4) -2 copies, t(10:Y), t(2;14)
2	141	del(17), del(3)
3	80	del(3), t(3:16)
4	76	del(1), del(2), del(4), del(18)
5	124	del(1), del(10), t(3;6), t(3:6)
6	74	del(5), del(14), del(16), t(2:Y), t(2:Y), t(Y:15)
7	77	del(6), break in chr 4
8	75	
10	66	t(5:7)
11	143	del(5) - 2 copies, del(14)- 2 copies, del (19), t(2;11), t(3;10), t(5:9), tandem (7:17)- 2 copies
12	154	del(12) - 2 copies

I **H1 P48 (84 ± 12 chr)**

Cell no.	Chromosome count	Chromosomal abnormalities
1	81	del(4), del(5), del(15), del(17), t(3:16), t(3:5), t(6:14), t(15:15),
2	82	del(14), t(3:16), t(8:11), t(15:15)
3	84	del(6), t(3;16), t(2:2) , t(4;11), tandem t(8q:8q)
4	86	t(3:16), t(3:16), t(3:8), t(8:15), tandem t(14:14), extensive double minutes
5	125	del(6) 2 copies, del(15) 2 copies, t(1:10)
6	62	t(3:16)
7	82	del(4), del(19), t(3:16), tandem t(8:8)
8	95	del(2), del(4) 2 copies, del(6), del(9), del(16)
9	78	del(2), del(4), t(3;16)
10	77	t(3:16)
11	85	extensive double minutes
12	86	del(14), del(17), t(3;16), t(13:13)
13	93	del(4) 2 copies, del(6), del(17), t(4:18), t(11:19), t(4:15), t(8:16)
14	86	del(2), del(3), del(15), t(3;16), t(4;8), t(8:15), t(12:19), tandem t(14;14)
15	82	t(3;16), t(3;16), t(3:16), t(13;14) tandem t(16:16)
16	81	del(3), del(13), del(15), t(3:14), tandem t(6:6), tandem t(11;19)
17	76	del(4), del(6), del(12), del(17), t(4:Y), t(5:17)
18	77	del(3), t(3;16), t(8:15), tandem t(14:14)

Appendix Figure S8. *Evolving numerical and segmental aneuploidy in immortalized mouse embryonic fibroblasts (MEFs).* Related to Fig. 4.

(A-C) DNA content measurement by propidium iodide staining in human and MEF cells. (A) The human breast cancer cell line Hs578T, which has many CNA aberrations (Neve *et al*, 2006), has substantially more DNA content than the immortalized but non-tumorigenic cell line MCF10A, which has a near diploid genome (Neve *et al*, 2006). (B-C) MEF cells show increased DNA content following immortalization and during subsequent evolution in culture. Three signature A cell lines were profiled at varying passage (P) numbers. These findings are consistent with past reports that, in contrast to immortalized MEFs, human mammary epithelial cells have an increased number of breakpoints and copy number alterations in tumorigenic but not immortalized cells (Garbe *et al*, 2014). Likewise, premalignant or hyperplasic human samples tend not to show signs of genomic instability (Chin *et al*, 2004; Ooi & Gomperts, 2015).

(D-G) Representative Spectral Karyotyping (SKY) images from the signature A H1 MEF subline at passage 25 (P25; cell no. 3 and 11; D-E) and P48 (cell no. 7; F-G). The total chromosome count for each image is shown. Nearly all chromosomes underwent whole chromosome gains, though some chromosomes experienced a higher degree of amplification (eg, chrs. 3 and 6). Chromosomal translocations are indicated by red arrows.

(H-I) Chromosome counts and chromosomal aberrations observed for all SKY scored metaphase spreads for the signature A H1 MEF subline at passage 25 (P25, H) and P48 (I). There was significant cell-to-cell heterogeneity in the number and type of chromosomes at passage 25, but clonal markers had begun to emerge by P48 (eg, t(3:16)). The average and standard deviation of the chromosome counts is shown. The average number of chromosomes decreased from P25 to P48 (from 105 to 84) as has been previously observed in immortalized MEF cells (Hao & Greider, 2004). We observed a substantial number of double minutes in 2 of 18 cells examined at P48. CGH profiles for H1 cells at passage numbers 25 and 48 are in Fig. 4A and Appendix Fig. S6A-B.

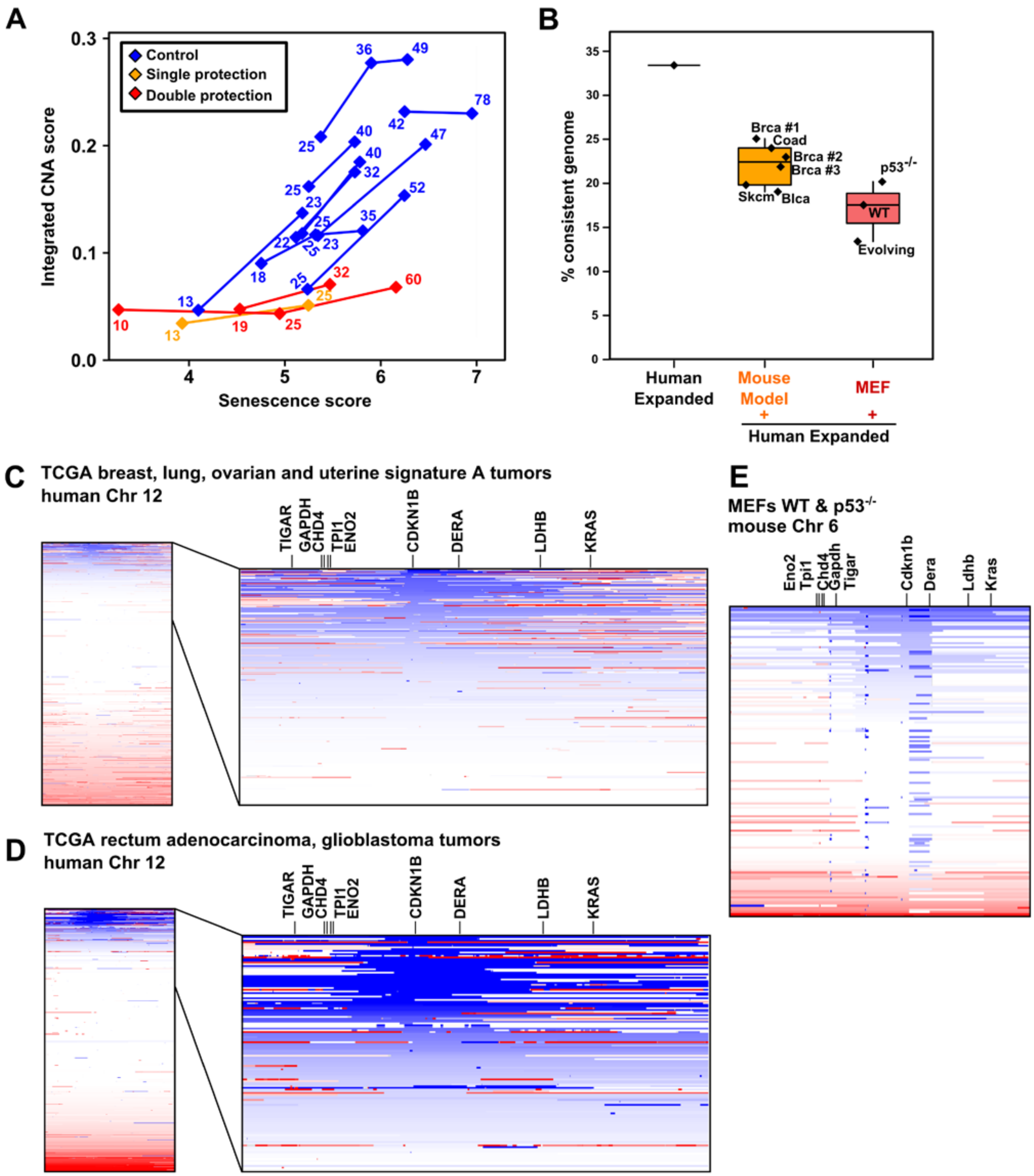
A
Control
Single protection
Double protection
Integrated CNA score
Senescence score
B
% consistent genome
Brca #1
Coad
Brca #2
Brca #3
Skcm
Blca
p53-/-
WT
Evolving
Human Expanded
Mouse Model
MEF
+
Human Expanded
C
TCGA breast, lung, ovarian and uterine signature A tumors
human Chr 12
TIGAR
GAPDH
CHD4
TPI1
ENO2
CDKN1B
DERA
LDHB
KRAS
D
TCGA rectum adenocarcinoma, glioblastoma tumors
human Chr 12
E
MEFs WT & p53-/-
mouse Chr 6
Eno2
Tpi1
Chd4
Gapdh
Tigar
Cdkn1b
Dera
Ldhb
Kras

Appendix Figure S9. *Characterization of the MEF CNA signatures.* Related to Fig. 4.

(A) The senescence score tended to increase during wild-type MEF line derivation (reflecting accumulated sub-optimal growth) and correlated with higher degrees of copy number alterations (integrated CNA score) observed at later passages. In comparison, ROS-protected sublines experienced less senescence and had lower amounts of copy number alterations at later passages. Single protection indicates protection by 3% O_2 culture conditions or by media supplementation with 250 u/ml Catalase. Double protection indicates that cells were cultured at 3% O_2 with catalase.

(B) The percentage of the genome that has consistent amplifications or deletions to human extended signature A tumors is similar when comparing the copy number alteration patterns in mouse tumor models (Brca #1-3, Blca, Skcm, and Coad) and MEF models (signature A WT and $p53^{-/-}$, and evolving).

(C-E) A positive-negative-positive selection pattern commonly observed around the tumor suppressor *CDKN1B* locus. In subsets of both human tumors and mouse models of cancer, the genomic region harboring *TIGAR-GAPDH-TPI-ENO2* was separated from the *LDHB-KRAS* region by a deletion region that includes the tumor suppressor *CDKN1B*. (C) TCGA breast, lung, ovarian and uterine signature A tumors, human chromosome (Chr) 12p region. (D) TCGA rectum adenocarcinoma and glioblastoma tumors. (E) MEFs, wild-type and $p53^{-/-}$, mouse chromosome 6 region**.** Tumor subsets are indicated by the expansion lines.

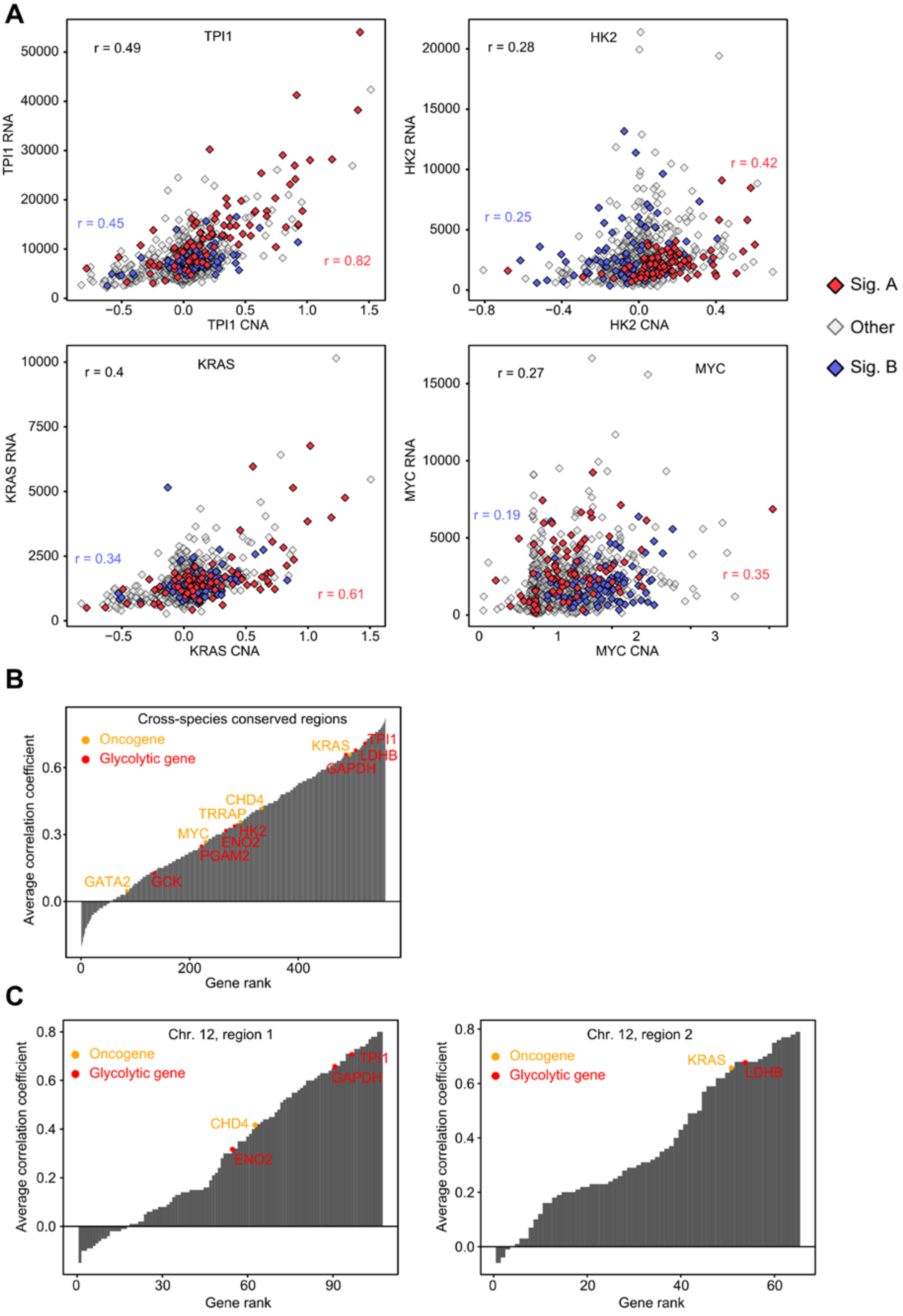
A
TPI1
r = 0.49
r = 0.45
r = 0.82
TPI1 RNA
TPI1 CNA
HK2
r = 0.28
r = 0.25
r = 0.42
HK2 RNA
HK2 CNA
KRAS
r = 0.4
r = 0.34
r = 0.61
KRAS RNA
KRAS CNA
MYC
r = 0.27
r = 0.19
r = 0.35
MYC RNA
MYC CNA
Sig. A
Other
Sig. B
B
Cross-species conserved regions
Oncogene
Glycolytic gene
KRAS
TPI1
LDHB
GAPDH
CHD4
TRRAP
MYC
HK2
ENO2
PGAM2
GATA2
GCK
Average correlation coefficient
Gene rank
C
Chr. 12, region 1
Oncogene
Glycolytic gene
TPI1
GAPDH
CHD4
ENO2
Average correlation coefficient
Gene rank
Chr. 12, region 2
Oncogene
Glycolytic gene
KRAS
LDHB
Average correlation coefficient
Gene rank

Appendix Figure S10. *Copy number alteration and RNA expression are correlated for both glycolytic genes and oncogenes in TCGA tumors.* Related to Fig. 4E-F.

(A) Correlation between DNA copy number level and RNA expression level for two glycolytic genes (*TPI1*, *HK2*) and two oncogenes (*KRAS*, *MYC*) in BRCA tumors. Signature A and B tumors (as defined by PCA of the CNA) are colored red and blue, respectively. Spearman correlation values for all tumors (black), signature A tumors (red) and signature B tumors (blue) are shown.

(B) Waterfall plot of the average Spearman rank correlation values across BRCA, LU and OV signature A tumors for all cross-species conserved genes shown in Figure 4E, bottommost row. Known oncogenes and glycolytic genes are noted with orange or red coloring, respectively.

(C) Waterfall plot of the average Spearman rank correlation values across BRCA, LU and OV signature A tumors for chromosome 12 regions 1 and 2 shown in Figure 4E, bottommost row. Known oncogenes and glycolytic genes are noted with an orange or red coloring, respectively.

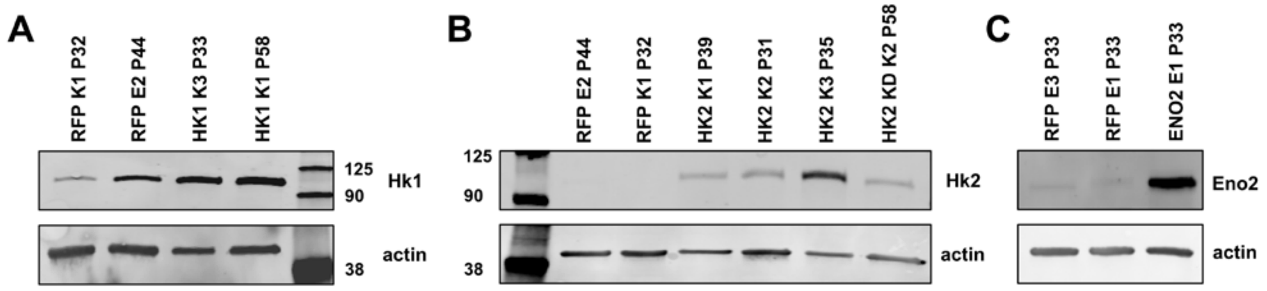

Appendix Figure S11. ___Genetic manipulation of signature A related metabolic genes.___ Related to Fig. 5.

(A-C) MEF lines expressing exogenous RFP, wild-type HK1, wild-type HK2, kinase-dead HK2 (D209A/D657A), or wild-type ENO2 were lysed and probed by immunoblotting for Hk1 (A), Hk2 (B) and Eno2 (C). Actin was included as an equal loading control. MEF sublines were each lysed at the passage number indicated by a capital letter P followed by the number. The lysates of the cells were matched when possible in passage number to the passage of the aCGH-profiled samples (passage of WB lysate collection/ passage of aCGH DNA collection): RFP K1 (P32/P34), RFP E2 (P44/P33), HK1 K3 (P33/P37), HK1 K1 (P58/P60), HK2 K1 (P39/P40), HK2 K2 (P31/P34), HK2 K3 (P35/P36), HK2 KD K2 (P58/P63), RFP E3 (P33/P33), RFP E1 (P33/P31), ENO2 E1 (P33/P25 and P33).

APPENDIX TABLE:

Tables EV1-5 provided as separate Excel files.

Appendix Table S1. *Trp53* sequencing in immortalized CD1 MEFs.

Sample	Signature	Mutation in Mm p53	Homologous mutation in Hs p53	Exons									
				**2**	**3**	**4**	**5**	**6**	**7**	**8**	**9**	**10**	**11**
H2 P25	B			wt	n.d.	wt	wt	wt	wt	wt	wt	wt	wt
E2 P54	B			wt	n.d.	wt	wt	wt	wt	n.d.	n.d.	wt	wt
A2 P25	B			wt	wt	wt	wt	wt	wt	wt	wt	wt	wt
A1 P25	B	**p.183 D>E†‡**	**p.186 D>E**	wt	n.d.	wt	**m.549 T>A**	n.d.	n.d.	n.d.	n.d.	n.d.	n.d.
B7 P99	B			wt	wt	wt	wt	wt	wt	wt	wt	wt	wt
D10 P36	mixed	**p.131 F>L**	**p.134 F>L**	n.d.	n.d.	wt	**m.393 C>A**	wt	wt	wt	wt	wt	n.d.
F1 P23	A	**p.R178***	**p.R181***	wt	n.d.	n.d.	**m.536 ***	n.d.	n.d.	n.d.	n.d.	n.d.	n.d.
A6 P40	A	**p.156 A>P**	**p.159 A>P**	wt	n.d.	wt	**m.466 G>C**	wt	wt	wt	n.d.	n.d.	n.d.
C8 P40	A	**p.270 R>C**	**p.273 R>C**	wt	wt	wt	wt	n.d.	wt	**m.808 C>T**	wt	n.d.	n.d.
A3 P25	A	**p.277 R>T**	**p.280 R>T**	wt	wt	wt	wt	wt	wt	**m.830 G>C**	wt	wt	n.d.
H1 P25	A	**p.129 K>T**	**p.132 K>T**	wt	n.d.	n.d.	**m.385 A>C**	wt	wt	n.d.	n.d.	n.d.	n.d.
C9 P42	A	**p.274 C>F**	**p.277 C>F**	n.d.	n.d.	wt	wt	wt	wt	**m.821 G>T**	wt	wt	n.d.

The cell lines and the passage number (P) at which p53 protein-coding exons were sequenced are indicated in the sample column. The homologous mutation in human p53 is indicated. †Non-severe mutation; ‡Heterozygous; *Nonsense mutation; wt, wild type sequence; n.d., not determined.

APPENDIX REFERENCES

Chin K, de Solorzano CO, Knowles D, Jones A, Chou W, Rodriguez EG, Kuo W-L, Ljung B-M, Chew K, Myambo K, Miranda M, Krig S, Garbe J, Stampfer M, Yaswen P, Gray JW & Lockett SJ (2004) In situ analyses of genome instability in breast cancer. *Nat. Genet.* **36:** 984–988

Garbe JC, Vrba L, Sputova K, Fuchs L, Novak P, Brothman AR, Jackson M, Chin K, LaBarge MA, Watts G, Futscher BW & Stampfer MR (2014) Immortalization of normal human mammary epithelial cells in two steps by direct targeting of senescence barriers does not require gross genomic alterations. *Cell Cycle* **13:** 3423–3435

Hao L-Y & Greider CW (2004) Genomic instability in both wild-type and telomerase null MEFs. *Chromosoma* **113:** 62–68

Neve RM, Chin K, Fridlyand J, Yeh J, Baehner FL, Fevr T, Clark L, Bayani N, Coppe J-P, Tong F, Speed T, Spellman PT, DeVries S, Lapuk A, Wang NJ, Kuo W-L, Stilwell JL, Pinkel D, Albertson DG, Waldman FM, McCormick F, Dickson RB, Johnson MD, Lippman M, Ethier S, Gazdar A & Gray JW (2006) A collection of breast cancer cell lines for the study of functionally distinct cancer subtypes. *Cancer Cell* **10:** 515–527

Ohgaki H & Kleihues P (2007) Genetic Pathways to Primary and Secondary Glioblastoma. *Am. J. Pathol.* **170:** 1445–1453

Ooi AT & Gomperts BN (2015) Molecular Pathways: Targeting Cellular Energy Metabolism in Cancer via Inhibition of SLC2A1 and LDHA. *Clin. Cancer Res.***:** clincanres.1209.2014
