# Supplementary material for: Recurrent patterns of DNA copy number alterations in tumors reflect metabolic selection pressures
Source: Mol Syst Biol. 2017 Feb 15;13(2):914. doi: 10.15252/msb.20167159 (PMC5327725; doi:10.15252/msb.20167159)
Supplement: Supplementary file 2 — Expanded View Figures PDF [file MSB-13-914-s002.pdf]

Expanded View Figures

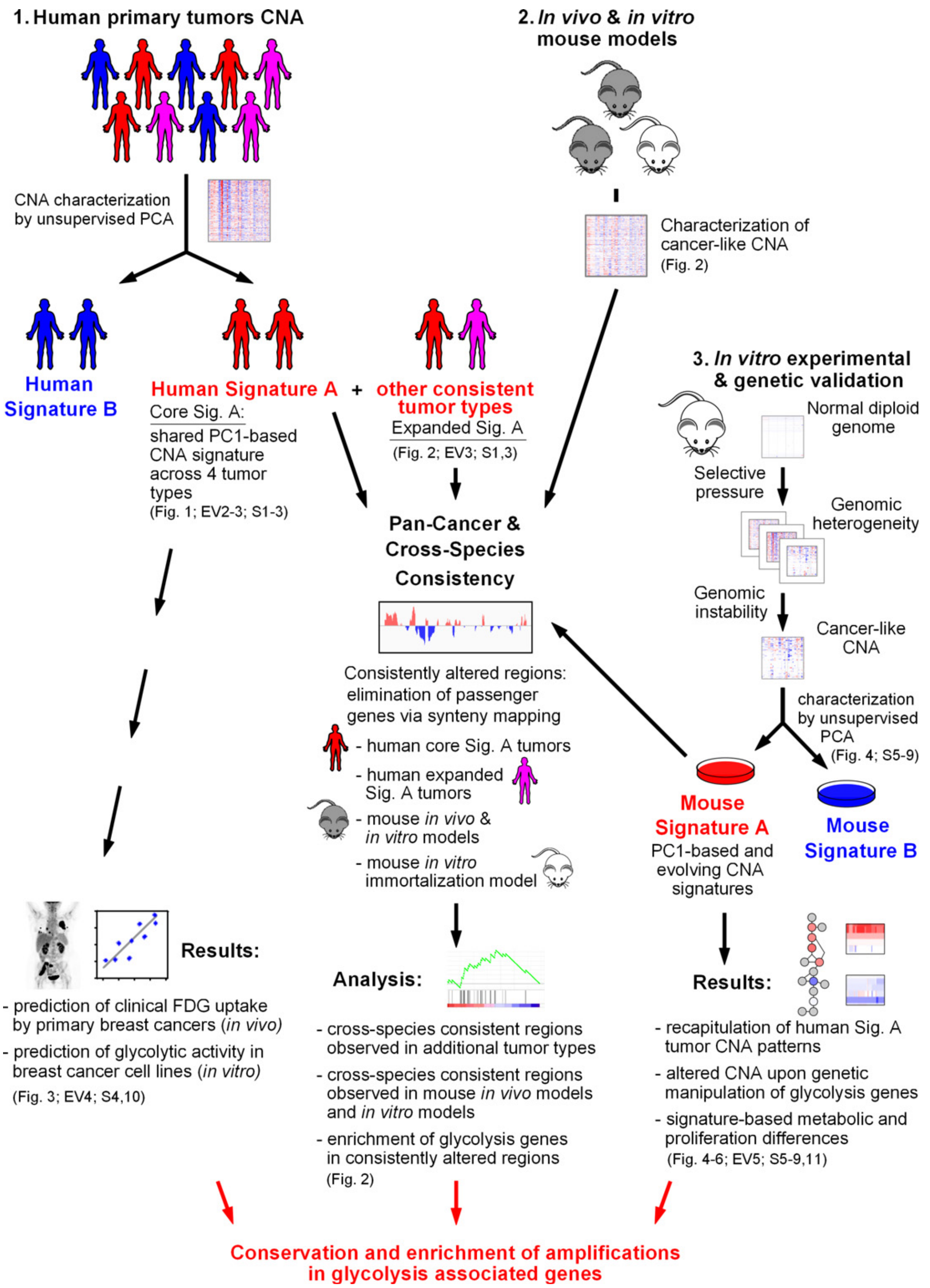

Figure EV1. Integration of patient and model system CNA in a pan-cancer and cross-species bioinformatic and experimental analysis.
Overall workflow for the analyses described in the Results section.

 © 2017 The Authors

Figure EV2. Principal component analysis of TCGA copy number data reveals a shared CNA signature enriched for p53 mutation (related to Fig 1).

A–C Copy number profiles of 359 lung (LU) squamous carcinomas (LUSC) and 368 lung adenocarcinomas (LUAD) (A), 583 ovarian serous cystadenocarcinoma (OV) (B), and 492 uterine corpus endometrial carcinomas (UCEC) (C) tumors sorted by their PC1 score. Signature A tumors are sorted to the top of the heatmap. PC1-ranked tumors were analyzed for enrichment of known mutations or molecular subtypes (e.g., basal/luminal in breast, see D for all subtypes tested). The red horizontal bars to the right of each tumor type indicate the tumor subtype (e.g., LUAD), or the presence of the indicated mutation (e.g., *TP53*). Normalized enrichment score and permutation *P*-value for each significantly enriched mutation or clinical subtype are shown below the tick plots.

D Enrichment analysis of molecular subtypes in PC-defined signatures A and B. The molecular subtypes tested were defined by the TCGA (The Cancer Genome Atlas Research Network, 2011, 2012a,b, 2013, 2014). Because LUAD and LUSC were combined into a single dataset LU, all LUSC and LUAD subtypes were associated with signatures A and B, respectively. Within LU, signature A was most associated with the classical subtype of LUSC, and signature B was most associated with the bronchioid subtype of LUAD. Permutation-based *P*-values are indicated.

E Amplification of *MDM2* and deletion of *CDKN2A* are mutually exclusive in signature B human BRCA. Each point represents a single patient sample. Samples are colored red or blue according to their membership in signatures A or B, respectively, with intermediate colors representing intermediate PC1 scores between signatures A and B. Samples colored white represent nearly diploid tumors, as indicated by the triangle in Fig 1B.

© 2017 The Authors

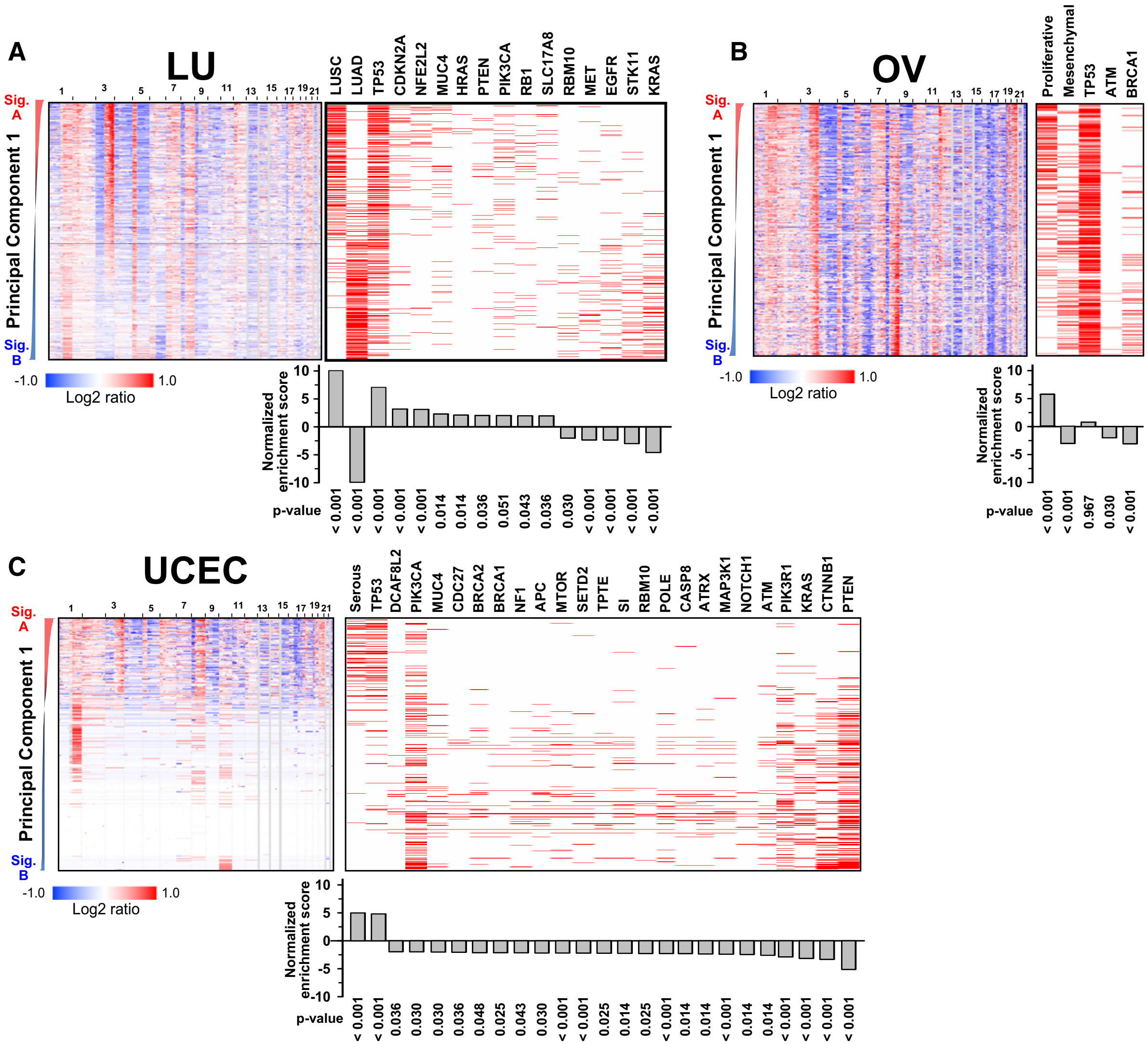

D

Molecular sub-type	NES	p-value	Signature B	Signature A
BRCA basal	7.38	< 0.001		
BRCA claudin-low	2.17	0.002		
BRCA HER2-enriched	-1.29	0.164		
BRCA luminal A	-2.62	< 0.001		
BRCA luminal B	-4.83	< 0.001		
BRCA luminal AB	-5.97	< 0.001		
LUSC classical	6.66	< 0.001		
LUSC primitive	3.97	< 0.001		
LUSC basal	3.77	< 0.001		
LUSC secretory	3.13	< 0.001		
LUAD magnoid	-2.74	< 0.001		
LUAD squamoid	-4.15	< 0.001		
LUAD bronchioid	-5.67	< 0.001		
OV proliferative	5.73	< 0.001		
OV immunoreactive	-2.03	0.007		
OV differentiated	-2.09	0.003		
OV mesenchymal	-2.70	< 0.001		
UCEC copy number high	7.12	< 0.001		
UCEC ultra-mutated	-2.11	0.004		
UCEC hyper-mutated	-2.91	< 0.001		
UCEC copy number low	-3.22	< 0.001		

E

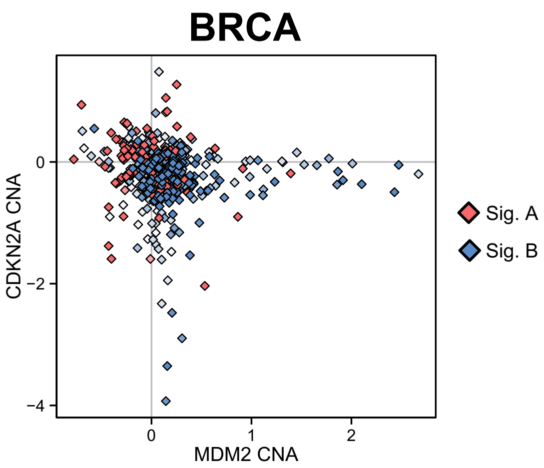

Figure EV2.

 © 2017 The Authors

Figure EV3. Hierarchical clustering confirms the existence of the shared pan-cancer CNA signatures across multiple tumor types, and distinct signature subtypes within tumor types (related to Fig 1).

Pan-cancer clustering of 3,037 copy number profiles using a balanced, random sampling of tumors from 15 tumor types. The dendrogram reveals the presence of a cluster containing multiple tumor types and enriched in PCA-defined signature A tumors (hypergeometric *P*-value = 1.1×10^{-70} for concordance between the PCA and hierarchical clustering results). There is also a cluster enriched in the generally less uniform PCA-defined signature B tumors (hypergeometric *P*-value = 4.5×10^{-12}), which contains other tumor types as well (e.g., SKCM). In the key, the individual tumor types and pan-cancer PC1 scores are indicated. Additionally, for the core signature A tumors (OV, BRCA, UCEC, and LU), tumor-specific subtypes (e.g., BRCA basal and luminal) and tumor type-specific PC1 scores are indicated. The gene loci CNA levels in the heatmap are ordered based on their chromosomal locations (columns).

© 2017 The Authors

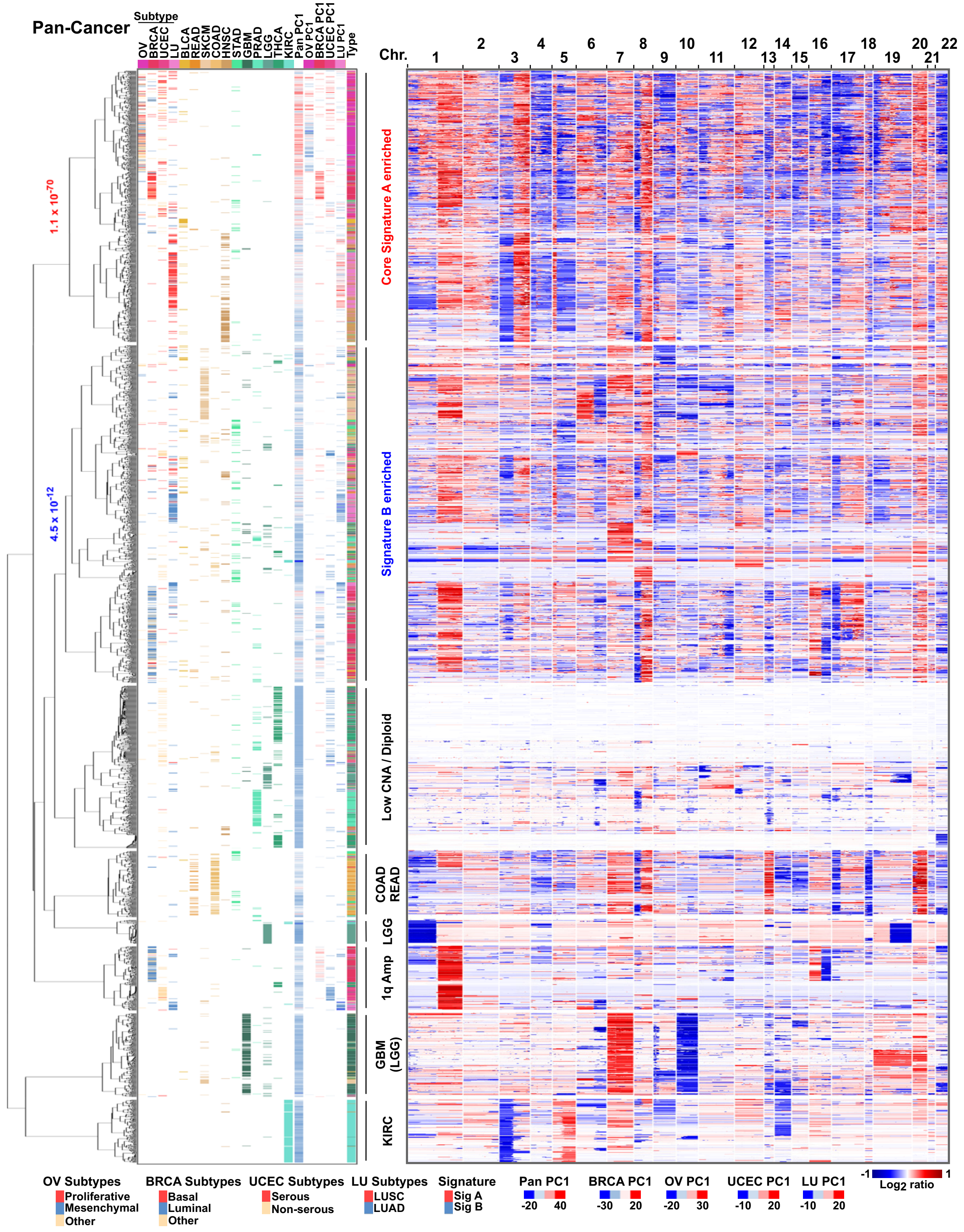

Figure EV3.

 © 2017 The Authors

Figure EV4. CNA-defined signature A tumors exhibit RNA expression signatures consistent with increased glycolytic activity (related to Fig 3).

A KEGG metabolism pathway enrichment analysis based on mRNA expression in BRCA and LU signature A and signature B tumors. RNA expression data are from BRCA and LU tumors with paired TCGA DNA and RNA data. To evaluate the similarity to BRCA tumors with high FDG-PET uptake, we included a gene set consisting of genes from the glycolysis and pentose phosphate pathway that were upregulated in FDG-high BRCA tumors (Palaskas *et al*, 2011). To combine BRCA and LU into one enrichment analysis, genes were ordered by their average rank in BRCA and LU tumor types. Enrichment scores for all KEGG metabolism pathways and for individual tumor types can be found in Table EV4.

B–D RNA expression signatures of high glycolysis (Palaskas *et al*, 2011) were used to predict the glycolytic phenotype of BRCA (B), LU (C), and OV (D) tumors with paired TCGA DNA and RNA data. UCEC tumors were excluded due to a lack of sufficient samples with paired RNA and DNA data. The color scale was normalized for each tumor type by the mean FDG score for tumors with integrated CNA score > 0.2. Non-normalized values are shown in the color legend and in (E).

E RNA-based WGV predictions of glycolysis show that BRCA and LU signature A tumors (top 10% PC1) are predicted to be more glycolytic than the tissue type-matched signature B tumors (bottom 10% PC1). Mann–Whitney U-test *P*-values are shown. Data are presented in box (median, first and third quartiles) and whisker (extreme value) plots.

F Spearman correlation values between RNA-based WGV glycolysis predictions and CNA-based PC1 values, calculated for different range windows of integrated CNA levels to control for the general increase in glycolysis predictions at increased levels of genomic instability. *P*-values shown are calculated by performing 1×10^6 permutations where the WGV scores were randomly shuffled, and the *P*-value was calculated as the percentage of random permutations that achieved a higher correlation value for any range window than did the experimentally observed relationship.

© 2017 The Authors

A

KEGG metabolism pathway gene set (n = 76)	Average gene rank BRCA+LU RNA SigA v. SigB: NES	Permutation p-value	FDR q-value
**FDG-PET High BRCA Palaskas et al.**	**2.52**	**0.0002**	**0.0073**
Pyrimidine metabolism	2.20	0.0010	0.0243
Pentose phosphate pathway	2.01	0.0051	0.0532
**Glycolysis & Pentose phosphate pathway**	**1.96**	**0.0068**	**0.0621**
Glycosphingolipid biosynthesis - lacto and neolacto series	1.86	0.0107	0.0868
Cysteine and methionine metabolism	1.83	0.0145	0.0962
**Core glycolysis**	**1.80**	**0.0156**	**0.0915**
Purine metabolism	1.73	0.0219	0.1230
...	...	...	...

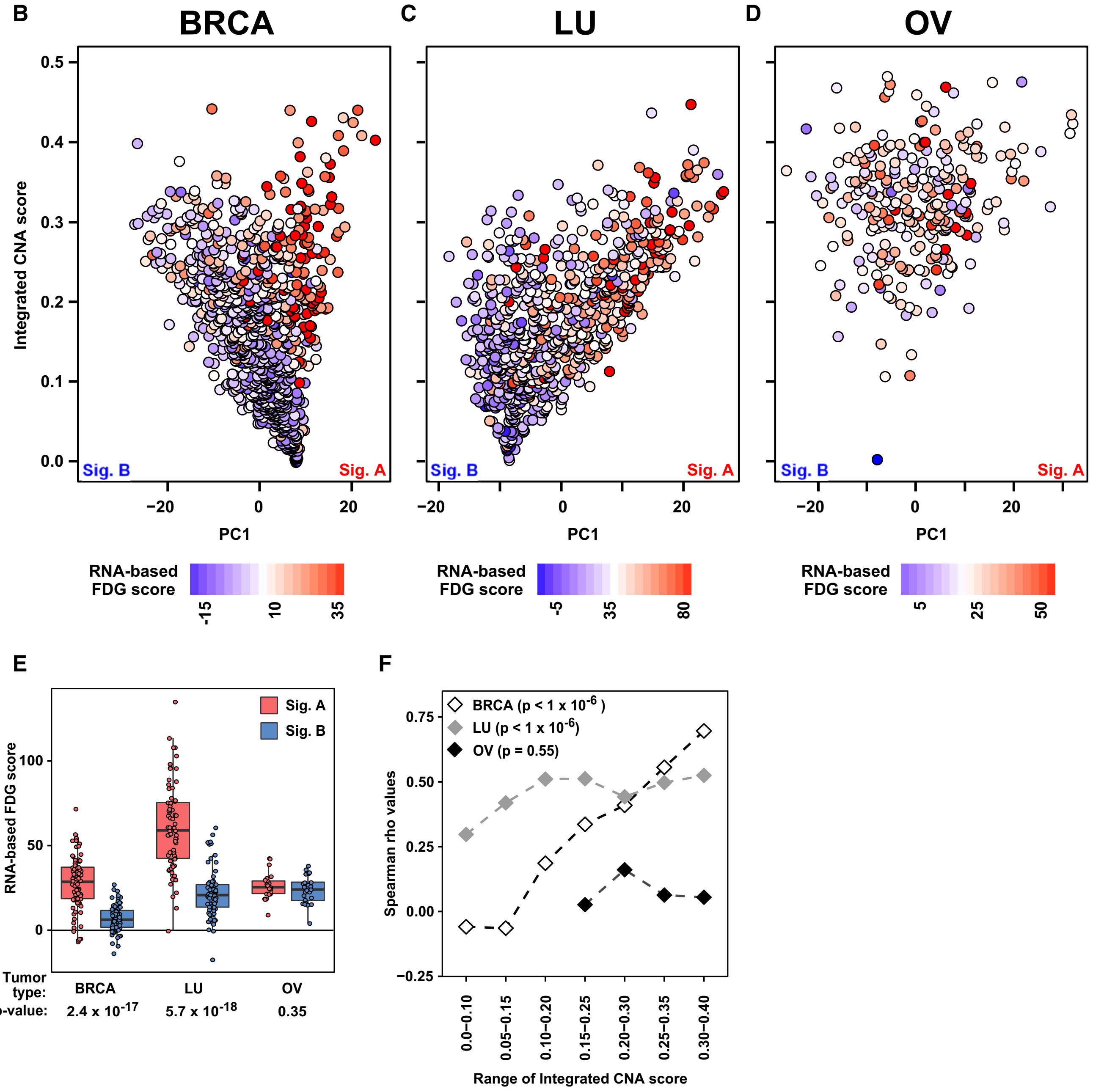

Figure EV4.

© 2017 The Authors

Figure EV5. Metabolic patterns are predictive of increased glycolysis in MEF lines (related to Fig 6).

A In cross-prediction tests, signature A-based predictions of signature A MEF line metabolic phenotypes perform better than signature B-based predictions. Glucose consumption was measured using a bioanalyzer.

B In cross-prediction tests, PC1 scores of signature A MEFs are more predictive of average growth fold change in signature A MEFs, while scores from signature B MEFs are more predictive in signature B MEFs.

C–G Mass spectrometry-based metabolite measurements. Samples are arranged left to right from the strongest signature B (blue) PC1 scores to the strongest signature A PC1 (red) scores. Sample D10 (purple) is a mixed signature A/B line. (C) Relative intracellular metabolite levels. (D) Isotopomer distributions for intracellular metabolites (M0, monoisotopic molecular weight). (E) Percent label for intracellular metabolites defined as percent of metabolite molecules with isotopomer mass greater than the monoisotopic molecular weight. Note, the high to low range for percent metabolite molecules with incorporated label varies for each metabolite and generally does not extend from 0 to 100%. (F) Relative metabolite consumption from and secretion into media normalized to initial media levels (i.e., cellular metabolite footprint). Here, positive and negative values indicate metabolite secretion into and consumption from the media, respectively. In (D–G), the correlation of each metabolic parameter with glucose consumption (as measured by a bioanalyzer, and as a positive variable, Fig 6A) is indicated (Pearson correlation, ρ). In the isotopomer cases (D), the summary metric of percent label is used for the correlation determination. Notably, cells with high glucose consumption rates tend to exhibit high consumption of serine, glycine, and glutamine; and high secretion of lactate (with concordant lactate secretion results obtained by bioanalyzer-based measurement of the same spent cell culture media). (G) Pathway schematics of differences in relative media levels (footprint, from F) between signature A and signature B MEFs. Each node is colored according the signal-to-noise (SNR) ratio. Red indicates higher post-culture media levels (lower consumption or higher secretion rates) in signature A MEFs, and blue indicates higher post-culture media levels in signature B MEFs. Error bars indicate standard deviations of biological replicates. Footprinting data were normalized to the integrated cell number of the time course of labeling (24 h), and intracellular metabolite concentrations were normalized to the number of cells present at the time of extraction. Full metabolomic data can be found in Table EV5.

© 2017 The Authors

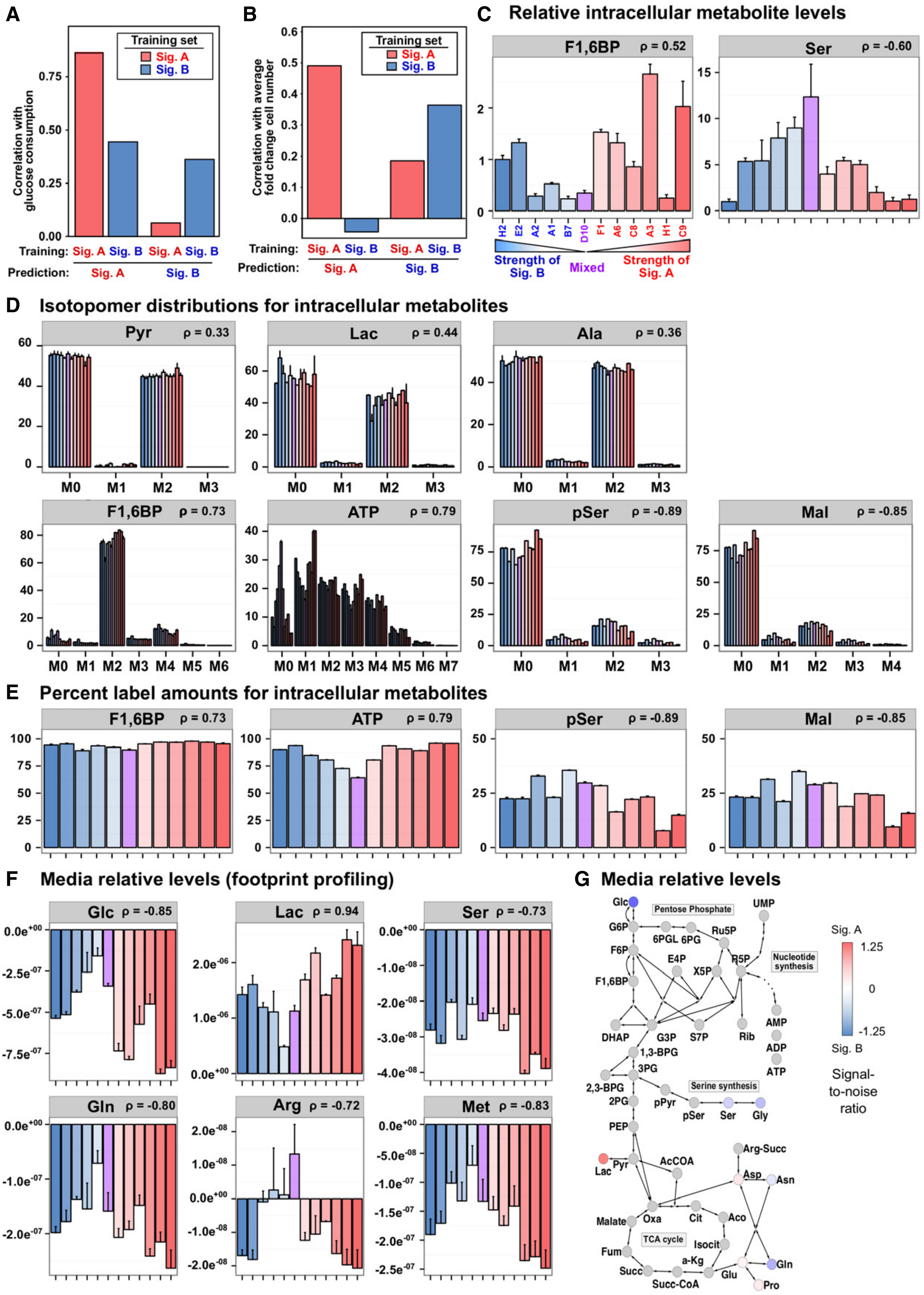

Figure EV5.

© 2017 The Authors
